# Antimicrobial Resistance of *Salmonella enteritidis* and *Salmonella typhimurium* Isolated from Laying Hens, Table Eggs, and Humans with Respect to Antimicrobial Activity of Biosynthesized Silver Nanoparticles

**DOI:** 10.3390/ani11123554

**Published:** 2021-12-14

**Authors:** Rasha M. M. Abou Elez, Ibrahim Elsohaby, Nashwa El-Gazzar, Hala M. N. Tolba, Eman N. Abdelfatah, Samah S. Abdellatif, Ahmed Atef Mesalam, Asmaa B. M. B. Tahoun

**Affiliations:** 1 Department of Zoonoses, Faculty of Veterinary Medicine, Zagazig University, Zagazig 44511, Egypt; rmmohamed@zu.edu.eg; 2 Department of Animal Medicine, Faculty of Veterinary Medicine, Zagazig University, Zagazig 44511, Egypt; 3 Department of Health Management, Atlantic Veterinary College, University of Prince Edward Island, Charlottetown, PE C1A 4P3, Canada; 4 Department of Infectious Diseases and Public Health, Jockey Club of Veterinary Medicine and Life Sciences, City University of Hong Kong, Kowloon, Hong Kong; 5 Department of Botany and Microbiology, Faculty of Science, Zagazig University, Zagazig 44519, Egypt; ns_elgsazzar@zu.edu.eg; 6 Department of Avian and Rabbit Medicine, Faculty of Veterinary Medicine, Zagazig University, Zagazig 44511, Egypt; halanabil85@gmail.com; 7 Department of Food Control, Faculty of Veterinary Medicine, Zagazig University, Zagazig 44511, Egypt; Enabil@vet.zu.edu.eg (E.N.A.); SSAbdelfattah@vet.zu.edu.eg (S.S.A.); abbadr@vet.zu.edu.eg (A.B.M.B.T.); 8 Department of Therapeutic Chemistry, National Research Centre (NRC), Dokki, Cairo 12622, Egypt

**Keywords:** *Salmonella*, antimicrobial agents, virulence genes, resistance genes, silver nanoparticles, expression

## Abstract

**Simple Summary:**

*Salmonella enterica* are common foodborne pathogens that cause gastrointestinal signs in a wide range of unrelated host species including poultry and humans. The overuse of antibiotics as therapeutic agents and growth promoters in the poultry industry has led to the emergence of multidrug-resistant (MDR) microorganisms. Thus, there is a need to find alternatives to conventional antibiotics. Recently, the biosynthesized silver nanoparticles (AgNPs) have shown an excellent antimicrobial activity. In this study, we investigated the antibacterial, antivirulent, and antiresistant activities of the biosynthesized AgNPs on the MDR and virulent *S. enteritidis* and *S. typhimurium* isolated from laying hens, table eggs, and humans. The obtained results indicated that AgNPs have the potential to be effective antimicrobial agents against MDR *S. enteritidis* and *S. typhimurium* and could be recommended for use in laying hen farms.

**Abstract:**

*Salmonella enterica* is one of the most common causes of foodborne illness worldwide. Contaminated poultry products, especially meat and eggs are the main sources of human salmonellosis. Thus, the aim of the present study was to determine prevalence, antimicrobial resistance profiles, virulence, and resistance genes of *Salmonella* Enteritidis (*S. enteritidis*) and *Salmonella* Typhimurium (*S.* Typhimurium) isolated from laying hens, table eggs, and humans, in Sharkia Governorate, Egypt. The antimicrobial activity of Biosynthesized Silver Nanoparticles (AgNPs) was also evaluated. *Salmonella* spp. were found in 19.3% of tested samples with laying hens having the highest isolation rate (33.1%). *S.* Enteritidis) (5.8%), and *S.* Typhimurium (2.8%) were the dominant serotypes. All isolates were ampicillin resistant (100%); however, none of the isolates were meropenem resistant. Multidrug-resistant (MDR) was detected in 83.8% of the isolates with a multiple antibiotic resistance index of 0.21 to 0.57. Most isolates (81.1%) had at least three virulence genes (*sopB*, *stn,* and *hilA*) and none of the isolates harbored the *pefA* gene; four resistance genes (*blaTEM*, *tetA*, *nfsA,* and *nfsB*) were detected in 56.8% of the examined isolates. The AgNPs biosynthesized by *Aspergillus niveus* exhibit an absorption peak at 420 nm with an average size of 27 nm. AgNPs had a minimum inhibitory concentration of 5 µg/mL against *S. enteritidis* and *S. typhimurium* isolates and a minimum bactericidal concentration of 6 and 8 µg/mL against *S. enteritidis* and *S. typhimurium* isolates, respectively. The bacterial growth and gene expression of *S. enteritidis* and *S. typhimurium* isolates treated with AgNPs were gradually decreased as storage time was increased. In conclusion, this study indicates that *S. enteritidis* and *S. typhimurium* isolated from laying hens, table eggs, and humans exhibits resistance to multiple antimicrobial classes. The biosynthesized AgNPs showed potential antimicrobial activity against MDR *S. enteritidis* and *S. typhimurium* isolates. However, studies to assess the antimicrobial effectiveness of the biosynthesized AgNPs in laying hen farms are warranted.

## 1. Introduction

*Salmonella enterica* are common foodborne pathogens, causing 93.8 million cases of gastroenteritis and 155,000 deaths worldwide and severe economic losses [1]. *Salmonella* Enteritidis (*S. enteritidis*) and *Salmonella* Typhimurium (*S. typhimurium*) are the dominant nontyphoidal serovars that cause mild gastrointestinal signs in a wide range of unrelated host species and severe infections in infants, the elderly, and immunocompromised individuals [2]. Nontyphoidal human salmonellosis is mostly associated with the ingestion of contaminated poultry meats and raw eggs [3]. However, table eggs get contaminated with *Salmonella* via horizontal transmission from the feces of infected laying hens, vertical transmission through the yolk, albumen, or eggshell membranes before oviposition and contamination of the eggshell after oviposition by infected environmental dusts [4].

Antibiotics have been extensively used in developing countries as therapeutic agents and growth promoters in laying hen farms as well as treatment for various human diseases, resulting in the emergence of antimicrobial resistance bacteria [5]. Antibiotic-resistant bacteria can be transmitted to humans either directly through the food chain or indirectly by transferring their antimicrobial resistance genes to human pathogens by mobile genetic elements associated with conjugative plasmids [6]. The presence of various virulence genes in the chromosome and plasmids of *Salmonella* plays a role in the pathogenesis of such bacteria inside the host [7]. *Salmonella* outer proteins (*sops*) and *hilA* virulence genes contribute to the invasion of host epithelial cells [8]. However, the plasmid-encoded fimbriae (*pefA*) gene is mediated through the adherence of *Salmonella* to intestinal epithelial cells [9], whereas the enterotoxin (*stn*) gene is responsible for enterotoxin production and diarrhea in the host [8]. *Salmonella* plasmid virulence (*spvs*) has relevance to *Salmonella* survival and replication inside the host [10]. Therefore, understanding the antibiotic resistance mechanisms will assist in the control and reduction in the spread of resistant bacteria. Furthermore, assessing the distribution of resistance genes in bacterial populations is an additional tool to understand the antimicrobial resistance epidemiology [11]. Thus, the development of an alternatives natural antimicrobial agent is needed.

Nanoparticles are one of the alternatives that could be used as antimicrobial agents in humans and animals [12]. Silver nanoparticles (AgNPs) are among the nanoparticles that have been used for many years in different applications including wound treatment. AgNPs physical, chemical, and biological properties depend on their size and shape [13]. AgNPs have good antimicrobial activity against bacteria, fungi, and viruses, with low cytotoxicity to mammalian cells due to their nanoscale size and various shapes [14]. The mechanism behind AgNPs’s actions includes their induction of cell death through generation of reactive oxygen species [15]. Furthermore, there are different factors that make AgNPs suitable for use as antimicrobial: (1) It is simple and safe to synthesize on a large scale with low cost [16]; (2) bacterial resistance to AgNPs is extremely rare; and (3) the surface of AgNPs can be easily modified and synthesized in various shapes [17]. Several studies have reported that AgNPs have antimicrobial activity against a variety of pathogens, including Salmonella enterica, *E. coli* O157:H7, Pseudomonas aeruginosa, Listeria monocytogenes, Klebsiella pneumoniae [18], and mastitis pathogens [19].

This study aimed to (1) determine the prevalence of *Salmonella* spp. in laying hens, table eggs, and humans, in Sharkia Governorate, Egypt; (2) detect the antimicrobial resistance profiles and virulence and resistance genes of *S. enteritidis* and *S. typhimurium* isolates; and (3) prepare and assess the antibacterial, antivirulent, and antiresistant activities of AgNPs biosynthesized by *Aspergillus niveus* (*A. niveus*) on the MDR and virulent *S. enteritidis* and *S. typhimurium* isolates.

## 2. Materials and Methods

### 2.1. Sample Collection

A total of 431 samples were collected from laying hen farms, egg retailers, and outpatient clinics in Sharkia Governorate, Egypt, between August 2019 and January 2020. Samples included 166 fecal swabs from laying hens, 165 table eggs, and 100 human stool samples. Aseptic techniques were strictly maintained during sample collection. Table eggs were individually packed in separate sterile plastic bags from egg retailers and brought to the laboratory. All human participants provided verbal or written consent to join the study.

### 2.2. Salmonella spp. Isolation

*Salmonella* was isolated from eggs and fecal samples as previously described Im, et al. [20], Yang, et al. [21]. The collected eggs were disinfected with 75% alcohol, the shell was removed and the yolks and whites were mixed. A 25 mL of the sample was added to 225 mL of sterile buffered peptone water (BPW; Oxoid, Basingstoke, Hampshire, UK) and then incubated at 37 °C for 24 h. A 100 μL of incubated BPW suspension was selectively enriched in 10 mL of Rappaport–Vassiliadis medium (Difco, Detroit, MI, USA) and incubated at 42 °C for 24 h. A loopful (10 μL) of enriched Rappaport–Vassiliadis medium was plated onto xylose lysine deoxycholate agar (Difco, Detroit, MI, USA) and incubated at 37 °C for 24 h. The suspected *Salmonella* isolates were kept frozen at −80°C in brain–heart infusion broth (Oxoid, Basingstoke, Hampshire, UK) supplemented with 15% glycerin (Synth^®^, São Paulo, Brazil). The presumptive colonies were picked up for purification on tryptone soya agar (Oxoid, Basingstoke, Hampshire, UK) and subjected to biotyping [22] and serotyping according to the Kauffmann–White scheme [23] to determine flagellar (H) and somatic (O) antigens using *Salmonella* antiserum (Denka Seiken, Tokyo, Japan).

### 2.3. Molecular Identification of Salmonella spp.

The extracted DNA was examined by PCR targeting *invA* gene using the QIAamp DNA Mini Kit (Qiagen, Hilden, Germany) according to the manufacturer’s guidelines [24]. Isolates identified as *Salmonella* spp. were subjected to identification of *sefA* gene (310 bp) for *S. enteritidis* [25] and *STM4495* gene (915 bp) for *S. typhimurium* [26]. PCR-confirmed *S. enteritidis* and *S.* Typhimurium isolates were screened to detect *sopB* [8], *stn* [9], *pefA* [9], *spvC* [8], and *hilA* [21] virulence genes. *S. typhimurium* ATCC 14028 and *S. enteritidis* ATCC 13076 were used as positive controls. However, *Escherichia coli ATCC 25922 was used as a negative control. The positive and negative controls were donated by the Biotechnology Unit, Reference Laboratory for Veterinary Quality Control on Poultry Production, Animal Health Research Institute, Dokki, Giza, Egypt, and were run alongside the tested isolates.*

### 2.4. Antimicrobial Susceptibility Test

Antimicrobial resistance of *S. enteritidis* and *S. typhimurium* isolates was determined using the Kirby–Bauer disk diffusion method on Mueller–Hinton Agar (MHA) (Oxoid, Hampshire, UK), in accordance with the guidelines of the Clinical and Laboratory Standards Institute [27]. The antibiotic disks used in this study were ampicillin (AMP, 10 μg), ampicillin/sulbactam (SAM, 20 μg), amoxicillin-clavulanate (AMC, 30 μg), cefotaxime (CTX, 30 μg), imipenem (IPM, 10 μg), gentamicin (GEN, 10 μg), tetracycline (TET, 30 μg), ciprofloxacin (CIP, 5 μg), nalidixic acid (NAL, 30 μg), trimethoprim-sulfamethoxazole (SXT, 25 μg), chloramphenicol (CHL, 30 μg), azithromycin (AZM, 15 μg), nitrofurantoin (NIT, 30 μg), and meropenem (MEM, 10 μg). The zone of inhibition, measured and compared with the world standards [27], was reported as resistant (R), intermediate (I), and sensitive (S). Isolates resistant to ≥3 different antimicrobial classes were considered MDR [28]. For all isolates, the multiple antibiotic resistance (MAR) index was determined using the formula: a/b (where “a” is the number of antimicrobial agents to which an isolate was resistant and “b” is the total number of antimicrobial agents tested) following the protocol designated by Krumperman [29].

*S. enteritidis* and *S. typhimurium* isolates with phenotypic resistance to specific antimicrobial agents were selected and tested for the presence of relevant resistance genes using PCR. The targeted genes included β-lactams (*blaTEM*) [30], tetracyclines (*tetA* and *tetB*) [31,32], and nitrofurans (*nfsA* and *nfsB*) [33].

### 2.5. Biosynthesis and Characterization of AgNPs

The AgNPs were biosynthesized from contaminated soil samples containing wastes from ceramics and photographic industries (10th of Ramadan City, Sharkia, Egypt) as described by [34]. Fungal isolate was screened to reduce the AgNo_3_ solution at 1 mM to AgNPs according to El-Gazzar and Rabie [35]. The available salt of AgNO_3_ was donated from the NanoTech Egypt (Dreamland, Egypt). The physical properties and concentrations of the biosynthesized AgNPs were investigated.

The extracted DNA of the most potent suspected fungal isolate producing AgNPs was molecularly identified at the Biology Research Unit of the Assiut University using Patho Gene-spin DNA/RNA Extraction Kit (iNtRON Biotechnology, Seongnam, Korea) following the manufacturer’s guidelines [36]. Isolates identified as *Aspergillus* spp. were further sequenced to target the same primers (SolGent, Daejeon, South Korea). The obtained sequence was analyzed using the BLAST tool and was placed in the GenBank (accession number MT319815). The nucleotide sequence was aligned with other sequences available at the GenBank to construct a phylogenetic tree using the MegAlign of DNASTAR program package (MegAlign 5.05, DNASTAR Inc., Madison, WI, USA). The recovered fungal isolate was characterized according to DeAlba-Montero, et al. [37] using different tools such as ultraviolet (UV)–visible spectrophotometer (T80 + UV Flash Spectrophotometer, PG Instruments Ltd., Wibtoft, UK); dynamic light scattering (DLS) system; transmission electron microscope (TEM) (JEOL.JEM.1010) at an accelerating voltage of 200 Kv; Fourier transform infrared spectroscopy (FTIR, Thermo Scientific Nicolet 6700 spectrometer) in the range of 400–4000 cm^−1^ and a resolution of 4 cm^−1^; and zeta potential analyzer (ZEN 1600, Malvern, UK).

### 2.6. Antimicrobial Activity of Biosynthesized AgNPs

The disk diffusion agar method was used to test the antimicrobial activity of biosynthesized AgNPs against *S. enteritidis* and *S. typhimurium* isolates [38]. A 10 µg of the prepared nanoparticles were paced on standard disks with a diameter of 6 mm (Padtanteb, Qods, Iran). The *S. enteritidis* and *S. typhimurium* isolates were spread on the Mueller–Hinton broth (MHB) (Merck, Darmstadt, Germany). The disks were placed on the agar plate and a sterile blank disk was used as control; then, the inhibition zone was measured after a 24 h incubation at 37 °C.

Minimum inhibitory concentration (MIC) and minimum bactericidal concentration (MBC) values of biosynthesized AgNPs were determined using tube dilution method as previously described Krishnan, et al. [39]. Bacterial inoculums were adjusted to match a 0.5 McFarland turbidity standard. A 100 µL of AgNPs with different concentrations (1, 2, 3, 4, 5, 6, 7, 8, 9, and 10 µg/mL) from each speed (4000, 8000, and 14,000 rpm) and a 100 µL from the tested organism were applied to 5 mL MHB and incubated at 37 °C with shaking for 24 h. Broth media with AgNPs inoculum was used as a positive control. The bacterial growth was monitored by measuring the mean OD at 600 nm.

The MBC was determined by coating the bacterial inoculum with different nanoparticle suspension concentrations into the MHA plate and then incubated at 37 °C for 24 h. The MBC value was defined as the lowest concentration, with no visible growths on the MHA plate.

### 2.7. Virulence and Resistance Genes Expression

#### 2.7.1. Bacterial Counting

The experiment used 2 MDR *Salmonella* isolates (*S. enteritidis* and *S. typhimurium* isolated during our study and were positive for most virulence and resistance genes). The bacterial inoculums were adjusted to 1.5 × 10^8^ Colony Forming Unite (CFU)/mL. The surface plating method was used for the bacterial count on an agar plate [40]. Bacterial inoculums were inoculated with a final concentration of 5 µg/mL followed by constant stirring to obtain a uniform colloidal nanoparticle suspension. Nanoparticle-free medium was used as a positive control, whereas the bacteria-free medium was used as a negative control. Each inoculated suspension was incubated at 37 °C for 0, 12, 24, 36, and 48 h. Bacterial growth inhibition was determined using the surface plating method by counting the number of CFUs on the plates. Bacteria and nanoparticle mixtures were prepared at different cultivation times. The amplification of the 16S rRNA gene was used to molecularly confirm *Salmonella* colonies. Bacterial cells were counted in triplicate and then the mean values and standard deviations were calculated.

#### 2.7.2. Quantitative Reverse Transcription PCR Analysis of Genes Expression

At each sampling time, 1 volume of the harvested bacterial culture was added to 1 volume of RNAprotect Bacteria Reagent (Qiagen, Hilden, Germany) following the manufacturer’s instructions. RNA isolation was implemented using QIAamp RNeasy Mini Kit (Qiagen, Hilden, Germany) according to the manufacturer’s guidelines. SYBR Green I-based real-time PCR with the specific primers for *sopB*, *stn,* and *hilA* virulence genes and *blaTEM*, *tetA,* and *nfsA* resistance genes (which are all present in the used 2 *Salmonella* isolates) was performed and the 16S rRNA gene was used as a housekeeping gene [21]. The primers were used in a 25 µL reaction containing 12.5 µL of 2× QuantiTect SYBR Green PCR Master Mix (QIAGEN), 0.25 µL of RevertAid Reverse Transcriptase (200 U/µL) (Thermo Fisher, Waltham, MA, USA), 0.5 µL of each primer (20 pmoL concentration), 8.25 µL of nuclease-free water, and 3 µL of RNA template. The reaction was performed in a Stratagene MX3005P real-time PCR machine where the amplification curves and cycle threshold (Ct) values were determined by Stratagene MX3005P software. The comparative Ct method was used to estimate the variations in RNA gene expression of the different samples by comparing Ct of each sample with that of the positive control. The ΔΔCt method was performed, according to Yuan, et al. [41].

### 2.8. Statistical Analysis

The R software (R Core Team, 2019; version 3.5.3) was used for the descriptive and statistical analysis. A heatmap was constructed based on the virulence and resistance genes and the antimicrobial susceptibility results using the R package “Complex heatmap” [42]. Nonmetric multidimensional scaling (nMDS) [43] was performed using the “metaMDS” function in “vegan” package to compare the dissimilarity of antimicrobial resistance profiles, using Bray–Curtis distance among isolates across all *Salmonella* spp. and within each species. The “corrplot” function in “corrplot” package was used to assess the correlation between the antimicrobial resistance and the presence of virulence and resistance genes. One-way analysis of variance was used to determine the significant difference between the bacterial counts and the fold change values of the gene’s expression at each storage time. Multiple comparisons between the means were assessed by the Tukey’s honestly significant difference test. *p* < 0.05 was considered statistically significant.

## 3. Results

### 3.1. Salmonella spp. Isolation and Identification

*Salmonella* spp. were identified in 83 of 431 examined samples (19.3%) and were mostly identified in samples from laying hens (33.1%) followed by humans (22%) and table eggs (3.6%) (Table 1). The most dominant *Salmonella* serotypes were *S. enteritidis* (5.8%) and *S. typhimurium* (2.8%), followed by *S. kentucky* (1.9%), *S. virchow* (1.6%), *S. tamale*, *S. inganda*, *S. wingrove*, *S. bargny* (1.2%, each), *S.* Anatum (0.9%), *S. tsavie*, *S. larochelle* (0.7%, each), and *S. apeyeme* (0.2%). On the other hand, only *S. apeyeme* was isolated from human samples (1 isolate), whereas *S. anatum*, *S. tsavie*, and *S. larochelle* were not identified (Table 1).

### 3.2. Antimicrobial Susceptibility Test

**Figure 1:**
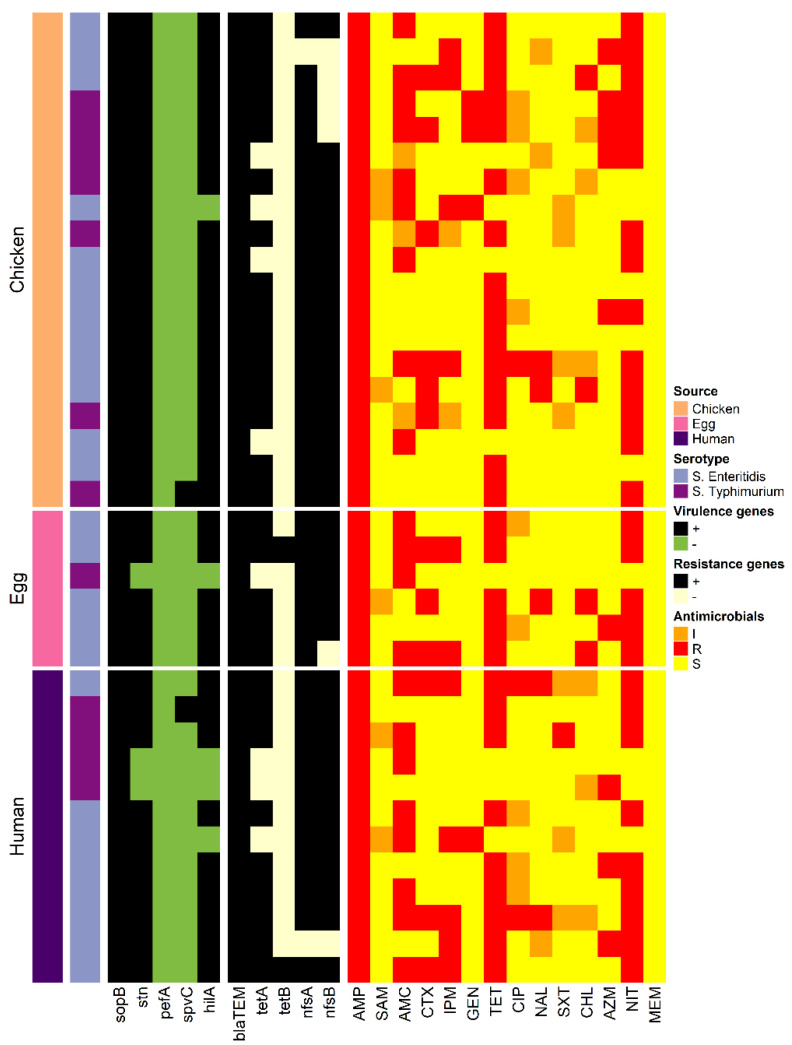
Heat map representation of the virulence and resistance genes and antimicrobial resistance profiles of *S. enteritidis* (*n* = 25) and *S. typhimurium* (*n* = 12) isolated from laying hens, table eggs, and humans.

The antimicrobial susceptibility profiles of the 25 *S. enteritidis* and 12 *S. typhimurium* isolates against 14 antimicrobial agents are presented in Table 2. All *S. enteritidis* and *S. typhimurium* isolates were resistant to AMP (100%) and sensitive to MEM (100%). In addition, *S. enteritidis* and *S. typhimurium* isolates exhibited high rates of resistance to tetracycline, NIT, and amoxicillin (Figure 1). The MDR was observed in 28 isolates (75.7%) with a MAR index ranging from 0.21 to 0.57.

**Figure 2:**
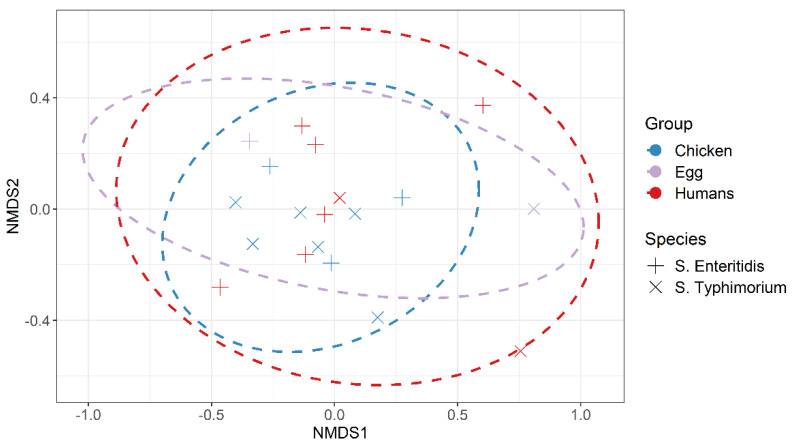
Non-metric multidimensional scaling ordination (NMDS) of antimicrobial-resistant of *S. enteritidis* and *S. typhimurium* isolated from laying hens, table eggs and humans.

The nMDS plot (Figure 2) shows that the antimicrobial profiles of *S. enteritidis* and *S. typhimurium* isolated from chickens, eggs, and humans’ samples were overlapped. The plot shows no evidence for clustering by isolate source.

### 3.3. Virulence and Resistance Genes

The presence of virulence and resistance-associated genes in *S. enteritidis* and *S. typhimurium* isolates is presented in Table 3. The *SopB* gene was identified in all *Salmonella* spp. isolates, whereas the *pefA* gene was not detected in any isolate. The *stn* virulence-associated gene was identified in all *S. enteritidis* and 75% of *S. typhimurium* isolates. The *hilA* gene was mostly identified in 94.6% of *Salmonella* isolates; however, the *SpvC* virulence gene was only detected in 5.4% of *S.* Typhimurium isolates. All *Salmonella* isolates (100%) harbored only 1 virulence gene and 34 isolates (91.9%) were positive for >1 virulence gene (Figure 1).

Five resistance genes (*blaTEM*, *tetA*, *tetB*, *nfsA* and *nfsB*) were also identified in *S. enteritidis* and *S. typhimurium* isolates (Table 3). The *blaTEM* gene was identified in all isolates and most isolates harbored *nfsA* (94.6%) and *nfsB* and *tetA* (83.8%, each) resistance genes. However, the *tetB* resistance gene was only recognized in two *S. enteritidis* isolates. Notably, five resistance profiles were exhibited in the study and most isolates (56.8%) were related to a profile carrying four resistance genes (*blaTEM*, *tetA*, *nfsA* and *nfsB*) (Figure 1).

**Figure 3:**
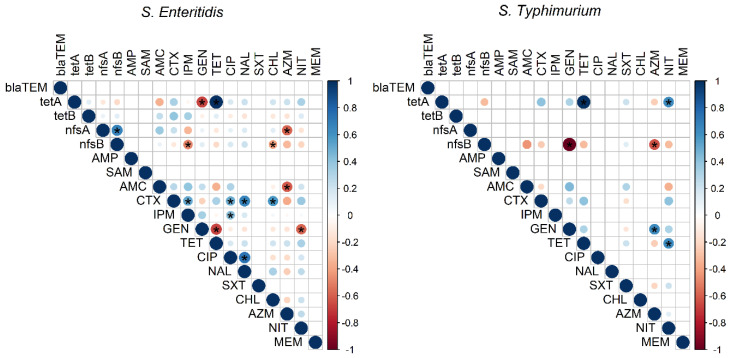
Correlation matrix showing the correlation between resistance phenotypes and genotypes among the examined *S. enteritidis* and *S. typhimurium* isolates recovered from laying hens, table eggs, and humans. The blue colour indicates a positive correlation and red shows a negative correlation. The asterisk (*) indicates significant at *p* < 0.001.

Significant correlations between resistance genes and antimicrobial agents are presented in Figure 3. There were significant positive correlations between resistance genes and their corresponding antimicrobial agents in most cases. The analysis also showed a significant negative correlation between resistance genes and antimicrobial agents other than the corresponding ones.

### 3.4. Characterization of Biosynthesized AgNPs

**Figure 4:**
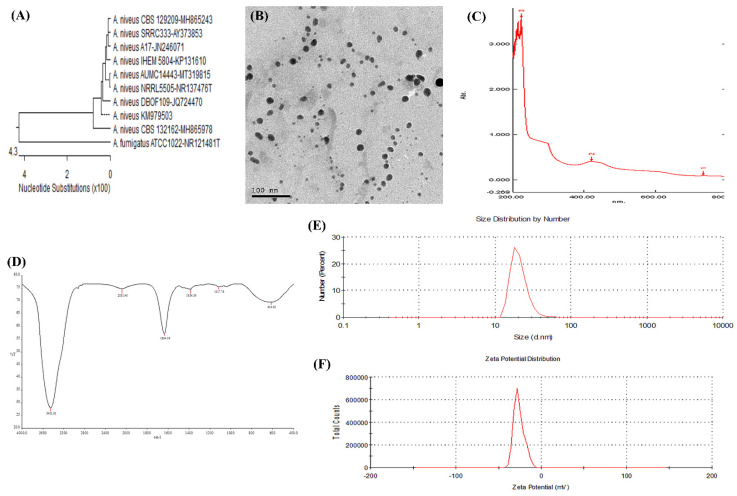
Characterization of AgNPs. (**A**) Phylogenetic analysis of the *Aspergillus niveus* (MT319815) used in the biosynthesis of AgNPs; (**B**) TEM micrographs and size distribution for silver (scale bar: 100 nm); (**C**) UV–Visible spectrum of AgNPs; (**D**) Fourier transform infrared spectrum showing the functional groups on the surface of AgNPs; (**E**) Dynamic light scattering analysis showing the highest peak at 27 nm and (**F**) Zeta-potential of AgNPs.

AgNPs were synthesized from *Aspergillus niveus* (*A. niveus*; MT319815), molecularly confirmed in the study targeting the 28S rRNA region (Figure 4A). AgNPs were oval, cubic, rod shaped, and well distributed without agglomeration at 6.49 nm using TEM microscopy (Figure 4B). The prepared AgNPs were characterized by the absorption peak of AgNPs at 420 nm using the UV–visible spectrum and an average particle size at 27 nm using DLS (Figure 4C). FTIR spectra of AgNPs confirmed the presence of various functional groups at 3451.81, 2081.40, 1634.19, 1384.39, 1117.76, and 614.02 cm^−1^. The peaks at 3451.81, 2081.40, 1634.19, and 614.02 cm^−1^ correspond to carbonyl residues, alcohol, nitrile, acid chloride, and alkene band C–C stretch in ring of CH3, stretch of alkyl halides and peptide bonds of proteins responsible for the synthesis of the AgNPs (Figure 4D). The bands at 3451.81, 1634.19, and 1384.39 cm^−1^ correspond to the binding vibrations of amide I and amide II of protein and hydroxyl O–H stretch of phenols with amine N–H stretchings. In addition, the band at 1117.76 cm^−1^ refers to one mononuclear aromatic. Dynamic light scattering analysis showed that the average particle size of the prepared AgNPs was at 27 nm (Figure 4E). The stability degree (Zeta Potential) of AgNPs showed a negative charge at −30.4 mv (Figure 4F).

### 3.5. Antimicrobial Activity of Biosynthesized AgNPs

The disk diffusion method was used to evaluate the antimicrobial activity of *S. enteritidis* and *S. typhimurium* isolates treated with AgNPs. The diameter of inhibition zones of AgNP (10 µg)-treated *S.* enteritidis and *S. typhimurium* isolates were 24 mm and 20 mm, respectively. The MIC for *Salmonella* treated with different concentrations (10, 9, 8, 7, 6, 5, 4, 3, 2, and 1 µg/mL) in this study was 5 µg/mL upon using the dilution method to *S. enteritidis* and *S. typhimurium* isolates, whereas MBC value was 6 µg/mL to *S. enteritidis* isolates and 8 µg/mL to *S. typhimurium* isolates.

**Figure 5:**
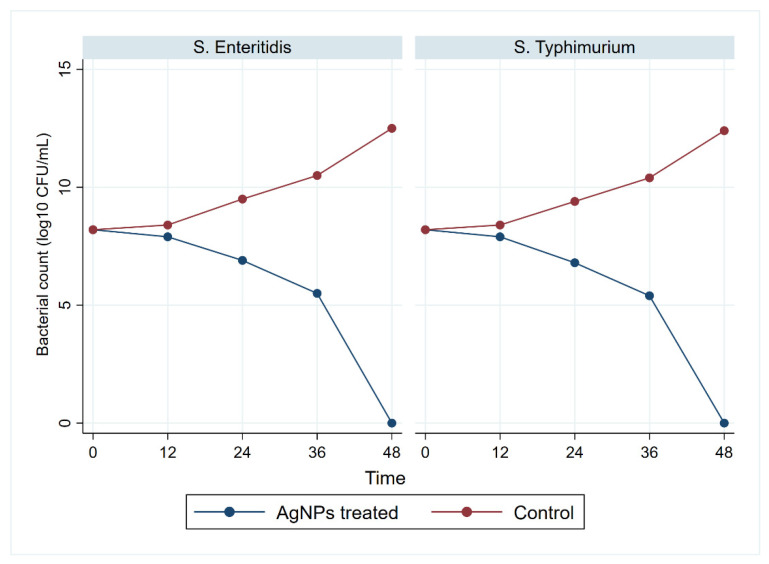
Bacterial growth of multidrug-resistant *S. enteritidis* and *S. typhimurium* treated with AgNPs. Bacterial counts were recorded at 0, 12, 24, 36, and 48 h post-treatment.

The selected MBC value for each strain was evaluated by assessing the bacterial growth (CFU/mL) at time intervals 12, 24, 36, and 48 h (Figure 5). The growth of *S. enteritidis* and *S*. *typhimurium* significantly (*p* < 0.05) declined at 12 h and reached complete inhibition at 48 h. In parallel, the control plates showed a significant increase in growth.

### 3.6. Antivirulent and Antiresistant Activity of Biosynthesized AgNP_s_

The expression patterns of virulence and resistance genes of *S. enteritidis* and *S. typhimurium* isolates treated with AgNPs at time intervals 6, 12, 24, and 36 h are presented in Table 4. The expression levels of virulence and resistance genes significantly decreased by time. Despite the loss of expression of the virulence genes at 36 h, the resistance genes showed instant expression.

## 4. Discussion

Chicken and eggs are considered one of the main reservoirs for zoonotic pathogens [44]. In this study, 12 different *Salmonella* serovars were identified in the examined samples with *Salmonella* spp. prevalence of 19.3%, and most of them were identified in laying hen samples (33.1%). The observed prevalence in laying hens was comparable to the 32.0% reported recently in Egypt [45] and the 33.3% in Colombia [46], but higher than the 1.02% and 10.4% reported in Spain [47] and Egypt [48], respectively. However, the prevalence of *Salmonella* spp. infection in laying hens was lower than the 59.3% that was reported previously in Korea [20]. Furthermore, the prevalence of *Salmonella* spp. infection in human samples (22%) was higher than the 4% that was reported previously in the same governorate [49]. In this study, the isolation rate of *Salmonella* spp. from egg content was low (3.6%) and lower than the 5.2% reported by Im, Jeong, Kwon, Jeong, Kang, and Lee [20] and the absence of *Salmonella* in egg content reported by Zubair, et al. [50]. The low incidence of *Salmonella* in egg content was caused by the egg’s complex system of membrane barriers and the albumen antibacterial effect [51]. Furthermore, *Salmonella* in egg contents may be attributed to contamination of eggshell by *Salmonella* infected poultry feces, which may penetrate the interior of eggs and grow during storage [52].

*S. enteritidis* and *S. typhimurium* were the most commonly isolated serotypes in this study, which was consistent with previous reports from Egypt [45] and Brazil [53]. The *S. enteritidis* and *S. typhimurium* isolates used in this study showed a high rate of antimicrobial resistance (100%) to at least one antimicrobial agent. Antimicrobial agents (AMP, TET, and NIT) were not effective against *Salmonella* isolates in this study. Our findings were comparable to those reported previously in South India [54] and Ghana [55]. The high rate of resistance reported in this study could be attributed to a number of factors, including the overuse of antimicrobial agents (over-the-counter antibiotics without a prescription) and the inappropriate use of antimicrobial agents as a growth promoter in animals, which could lead to the emergence of resistant bacteria in both animals and humans through direct contact or through the food chain [56]. All *Salmonella* isolates in this study were sensitive to SAM and MEM, which was consistent with previous Egyptian reports [57,58], and was accredited to the limited use of these antimicrobial agents in commercial chicken farms.

Previously, MDR from various sources has been identified in *Salmonella* [57,59], making these antimicrobial agents ineffective in humans and poultry [60]. In the current study, 75.7% of *Salmonella* isolates displayed MDR, which is higher than the prevalence of MDR *Salmonella* isolated from chicken in the USA [61] and China [62,63]. Despite this, the prevalence of MDR *Salmonella* in this study was lower than the 100% previously reported in chicken in Egypt [57] and 92.9% in North India [59]. The resistant isolates were not evenly distributed in laying hens, eggs, and humans. However, these isolates were overlapped, reflecting the high public health impact of MDR isolates and the need for new antimicrobial agents to treat human salmonellosis [64].

The presence of both virulence and antimicrobial resistance genes affects the bacteria’ pathogenicity [8]. The emergence of MDR strains of *Salmonella* is primarily due to genetic factors that enhance their survival by retaining drug resistance genes [65]. Of the confirmed *S. enteritidis* and *S. typhimurium* isolates, 81.1% harbored three virulence genes (*sopB*, *stn,* and *hilA*), which was similar to the previous study in Malaysia [66]. None of the isolates harbored *pefA* gene in our study, which was similar to a previous study in Egypt [45]. In contrast to Thung, Radu, Mahyudin, Rukayadi, Zakaria, Mazlan, Tan, Lee, Yeoh, and Chin [66], who reported the absence of *spvC* gene *Salmonella* isolates, the *spvC* was identified in 5.4% of *S. enteritidis* and *S. typhimurium* isolates.

In the present study, *Salmonella* harbors a range of antibiotic resistance genes located on mobile genetic elements that distribute resistance characteristics to other serovars and other different bacteria [59]. All isolates had *blaTEM* resistance genes and most of them had *tetA*, *nfsA,* and *nfsB* resistance genes, which was similar to *Salmonella* isolates from chickens in Egypt [57] and North India [59]. The *TetB* was not identified in previous studies, but *tetA* was the most common, highlighting its resistance role to tetracyclines [59,67].

There is currently a need for more research to find novel materials that inhibit antimicrobial-resistant variants [68]. Therefore, the development of natural antimicrobial agents such as AgNPs may be an alternative way to overcome MDR bacteria. In our study, AgNPs were biosynthesized from molecularly confirmed *A. nivus*. in line with the previous study by Elgazzar and Ismail [69], the peak of AgNPs using UV–visible spectra was witnessed at 420 nm. The small particle size of AgNPs (27 nm) with a single peak indicates the suitable quality of the biosynthesized nanoparticles and ideal inhibitory effects on bacterial growth [70]. The small particle size allows AgNPs to bind to the cell wall and easily penetrate the bacteria cell, enhancing their antimicrobial activity against the bacteria owing to their larger surface area and greater interaction [71]. The TEM microscopy indicated that biosynthesized AgNPs were well dispersed in the solution without agglomeration, explaining that fungal filtrates contain various biomolecules used to cap and stabilize the agglomeration of AgNPs [35]. FTIR measurements of biosynthesized AgNPs have verified different functional groups of biomolecules and capped proteins, excreted by the fungus itself, encapsulated nanoparticles, and increased stability; associated proteins may help to mineralize precursor salts [69]. The higher negative charge of the AgNPs as measured by the zeta potential confirms the synthesized particles’ repulsion, resulting in stability and monodispersity of the synthesized AgNPs solution [72].

In this study, a clear zone was found to exist around the AgNP disk, suggesting that the biosynthesized AgNPs possessed a potent antibacterial effect against the growth of MDR bacteria, causing a reduction in bacterial number [73,74]. To evaluate the effect of AgNPs against *Salmonella*, MIC and MBC values were examined in our study, showing the extent of variation with Loo, et al. [74], who reported that the MIC value of *S. enteritidis* and *S. typhimurium* was 3.9 mg/mL (each) and the MBC values for *S. enteritidis* and *S. typhimurium* were 3.9 mg/mL and 7.8 mg/mL, respectively.

The bacterial growth inhibition of AgNPs occurs because AgNPs interfere with sulfur found in biomolecules on the bacterial membrane, attacking the bacterial genome and resulting in bacterial death [75]. In this study, a significant decrease was found in the number of *Salmonella* isolates after different storage time and the growth was completely absent after 48 h of incubation; however, Tanhaeian and Ahmadi [76] reported a decline in the bacterial growth treated with 25 µg/mL and 50 µg/mL of AgNP after 10 h and the growth level dropped to almost zero.

Besides exploring the effect of the biosynthesized AgNPs on the growth inhibition of *Salmonella*, we investigated their impact on the expression of genes that are essential to the virulence and resistance of the pathogen. The results emphasized that biosynthesized AgNPs at a concentration of 5 µg significantly reduce gene expression, as previously reported [76]. The adverse effect of the biosynthesized AgNPs on virulence and resistance genes expression contributes to effective preventative and potent antimicrobial drugs against bacterial infection [76]. AgNPs are broad-spectrum antimicrobial agents that have the same effect on all Gram-negative bacteria strains. However, the concentration of the used AgNPs and bacteria class, nanoparticle size, synthesis method, and physical characteristics of nanoparticles are key factors that can affect the results across studies.

## 5. Conclusions

In the present study, *S. enteritidis* and *S. typhimurium* were the most common *Salmonella* serovars isolated from laying hens, table eggs, and humans in Egypt. Moreover, most of the recovered *Salmonella* isolates exhibited MDR, which poses a possible risk to consumers in Egypt. The findings of this study support that AgNPs have the potential to be effective antimicrobial agents against MDR *S. enteritidis* and *S. typhimurium* and could be recommended for use in laying hen farms. However, further studies are required to develop and design a safe AgNPs antimicrobial for laying hen farms.

## Figures and Tables

**Table 1:** Serotypes and pathotypes of *Salmonella* spp. isolated from laying hens, table eggs and humans.

Serotypes	Pathotypes	No. (%) of Isolates			Total (*n* = 431)
		Laying Hens (*n* = 166)	Table Eggs (*n* = 165)	Humans (*n* = 100)	
*S.* *enteritidis*	D1; O:1, 9, 12; H:g, m:−	12 (7.2)	5 (3.0)	8 (8.0)	25 (5.8)
*S.* *typhimurium*	B; O:1, 4, 5, 12; H:i:1, 2	7 (4.2)	1 (0.6)	4 (4.0)	12 (2.8)
*S.* *kentukey*	C3; O:8, 20; H:i:z6	5 (3.01)	0 (0.0)	3 (3.0)	8 (1.9)
*S.* *virchow*	C1; O:6, 7, 14; H:r:1, 2	5 (3.01)	0 (0.0)	2 (2.0)	7 (1.6)
*S.* *tamale*	C3; O:8, 20; H:Z29:e, n, Z15	4 (2.4)	0 (0.0)	1 (1.0)	5 (1.2)
*S.* *inganda*	C1; O:6, 7; H:Z10:1, 5	4 (2.4)	0 (0.0)	1 (1.0)	5 (1.2)
*S.* *wingrove*	C2; O:6, 8; H:c:1, 2	4(2.4)	0 (0.0)	1 (1.0)	5 (1.2)
*S.* *bargny*	C3; O:8, 20; H:i:1, 5	4 (2.4)	0 (0.0)	1 (1.0)	5 (1.2)
*S.* *anatum*	E1; O:3, 10; H:e, h:1, 6	4 (2.4)	0 (0.0)	0 (0.0)	4 (0.9)
*S.* *tsavie*	B; O:4,5; H:i:e, n, z15	3 (1.8)	0 (0.0)	0 (0.0)	3 (0.7)
*S.* *larochell*	C1; O:6, 7; H:e, h:1, 2	3 (1.8)	0 (0.0)	0 (0.0)	3 (0.7)
*S.* *apeyeme*	C3; O:8, 20; H:Z38:−	0 (0.0)	0 (0.0)	1 (1.0)	1 (0.2)
Total		55 (33.1)	6 (3.6)	22 (22)	83 (19.3)

**Table 2:** The antimicrobial resistance profile of *S. enteritidis* (*n* = 25) and *S. typhimurium* (*n* = 12) isolated from laying hens, table eggs, and humans.

Antimicrobials Class	Antimicrobials	*S. enteritidis* Isolates (%)			*S. typhimurium* Isolates (%)		
		R	I	S	R	I	S
Penicillin	Ampicillin (AMP)	25 (100)	0 (0.0)	0 (0.0)	12 (100)	0 (0.0)	0 (0.0)
	Ampicillin/Sulbactam (SAM)	0 (0.0)	4 (16.0)	21 (84.0)	0 (0.0)	2 (16.7)	10 (83.3)
	Amoxicillin-Clavulanate (AMC)	15 (60.0)	0 (0.0)	10 (40.0)	6 (50.0)	3 (25.0)	3 (25.0)
Cephalosporine	Cefataxime (CTX)	9 (36.0)	0 (0.0)	16 (64.0)	3 (25.0)	0 (0.0)	9 (75.0)
Carbapenems	Imipenem (IPM)	11 (44.0)	0 (0.0)	14 (56.0)	0 (0.0)	2 (16.7)	10 (83.3)
Aminoglycosides	Gentamicin (GEN)	2 (8.0)	0 (0.0)	23 (92.0)	2 (16.7)	0 (0.0)	10 (83.3)
Tetracyclines	Tetracycline (TET)	22 (88.0)	0 (0.0)	3 (12.0)	8 (66.7)	0 (0.0)	4 (33.3)
Quinolones	Ciprofloxacin (CIP)	3 (12.0)	6 (24.0)	16 (64.0)	0 (0.0)	3 (25.0)	9 (75.0)
	Nalidixic acid (NAL)	5 (20.0)	2 (8.0)	18 (72.0)	0 (0.0)	1 (8.3)	11 (91.7)
Sulphonamides	Trimethoprim-Sulfamethoxazole (SXT)	0 (0.0)	5 (20.0)	20 (80.0)	1 (8.3)	2 (16.7)	9 (75.0)
Phenicols	Chloramphenicol (CHL)	4 (16.0)	3 (12.0)	18 (72.0)	0 (0.0)	3 (25.0)	9 (75.0)
Macrolides	Azithromycin (AZM)	5 (20.0)	0 (0.0)	20 (80.0)	4 (33.3)	0 (0.0)	8 (66.7)
Nitrofurans	Nitrofurantoin (NIT)	20 (80.0)	0 (0.0)	5 (20.0)	8 (66.7)	0 (0.0)	4 (33.3)
Carbapenem	Meropenem (MEM)	0 (0.0)	0 (0.0)	25 (100)	0 (0.0)	0 (0.0)	12 (100)

R = resistant, I = intermediate resistance, S = sensitive.

**Table 3:** Virulence and resistance genes and antimicrobial resistance patterns of *S. enteritidis* (*n* = 25) and *S. typhimurium* (*n* = 12) isolated from laying hens, table eggs and humans.

Source	No. of Isolates	Virulence Genes					Resistance Genes					No. of Ab	Resistance Patterns	MARIndex
		*sopB*	*stn*	*pefA*	*spvC*	*hilA*	*blaTEM*	*tetA*	*tetB*	*nfsA*	*nfsB*			
(**I**) ***S. enteritidis***														
Human	2	+	+	−	−	+	+	+	−	+	+	8	AMP, AMC, CTX, IPM, TET, CIP, NA, NIT	0.57
Chicken	1	+	+	−	−	+	+	+	−	+	+	8	AMP, AMC, CTX, IPM, TET, CIP, NA, NIT	0.57
Chicken	1	+	+	−	−	+	+	+	−	+	−	7	AMP, AMC, CTX, IPM, TET, NA, NIT	0.50
Egg	1	+	+	−	−	+	+	+	−	+	−	7	AMP, AMC, CTX, IPM, TET, NA, NIT	0.50
Egg	1	+	+	−	−	+	+	+	+	+	+	6	AMP, AMC, CTX, IPM, TET, NIT	0.43
Human	1	+	+	−	−	+	+	+	+	+	+	6	AMP, AMC, CTX, IPM, TET, NIT	0.43
Egg	1	+	+	−	−	+	+	+	−	+	+	6	AMP, CTX, IPM, TET, NAL, NIT	0.43
Chicken	1	+	+	−	−	+	+	+	−	+	+	6	AMP, CTX, IPM, TET, NAL, NIT	0.43
Chicken	1	+	+	−	−	+	+	+	−	−	−	5	AMP, IPM, TET, AZM, NIT	0.36
Human	1	+	+	−	−	+	+	+	−	−	−	5	AMP, IPM, TET, AZM, NIT	0.36
Chicken	1	+	+	−	−	+	+	+	−	+	+	4	AMP, AMC, TET, NIT	0.29
Egg	1	+	+	−	−	+	+	+	−	+	+	4	AMP, AMC, TET, NIT	0.29
Human	2	+	+	−	−	+	+	+	−	+	+	4	AMP, AMC, TET, NIT	0.29
Egg	1	+	+	−	−	+	+	+	−	+	+	4	AMP, TET, AZM, NIT	0.29
Chicken	1	+	+	−	−	+	+	+	−	+	+	4	AMP, TET, AZM, NIT	0.29
Human	1	+	+	−	−	+	+	+	−	+	+	4	AMP, TET, AZM, NIT	0.29
Chicken	1	+	+	−	−	−	+	−	−	+	+	4	AMP, AMC, IPM, GEN	0.29
Human	1	+	+	−	−	−	+	−	−	+	+	4	AMP, AMC, IPM, GEN	0.29
Chicken	2	+	+	−	−	+	+	−	−	+	+	3	AMP, AMC, NIT	0.21
Chicken	3	+	+	−	−	+	+	+	−	+	+	2	AMP, AMC	0.14
(**II**) ***S. typhimurium***														
Chicken	1	+	+	−	−	+	+	+	−	+	−	7	AMP, AMC, CTX, GEN, TET, AZM, NIT	0.50
Chicken	1	+	+	−	−	+	+	+	−	+	−	6	AMP, AMC, GEN, TET, AZM, NIT	0.43
Human	1	+	+	−	−	+	+	+	−	+	+	5	AMP, AMC, TET, SXT, NIT	0.36
Chicken	2	+	+	−	−	+	+	+	−	+	+	4	AMP, CTX, TET, NIT	0.29
Human	1	+	+	−	+	+	+	+	−	+	+	3	AMP, TET, NIT	0.21
Chicken	1	+	+	−	+	+	+	+	−	+	+	3	AMP, TET, NIT	0.21
Chicken	1	+	+	−	−	+	+	−	−	+	+	3	AMP, AZM, NIT	0.21
Chicken	1	+	+	−	−	+	+	+	−	+	+	3	AMP, AMC, TET	0.21
Human	1	+	−	−	−	−	+	−	−	+	+	2	AMP, AMC	0.14
Egg	1	+	−	−	−	−	+	−	−	+	+	2	AMP, AMC	0.14
Human	1	+	−	−	−	−	+	−	−	+	+	2	AMP, AZM	0.14

**Table 4:** Virulence and resistance genes expression of multidrug-resistant *S. enteritidis* and *S. typhimurium* treated with AgNPs.

Storage Time (Hours)	Virulence Genes Expression			Resistance Genes Expression		
	*SopB*	*stn*	*hilA*	*blaTEM*	*tetA*	*nfsA*
(**I**) ***S. enteritidis***						
**0**	1.0 ± 0.0	1.0 ± 0.0	1.0 ± 0.0	1.0 ± 0.0	1.0 ± 0.0	1.0 ± 0.0
**6**	0.71 ± 0.015	0.73 ± 0.025	0.69 ± 0.015	0.85 ± 0.02	0.78 ± 0.01	0.83 ± 0.006
**12**	0.45 ± 0.015	0.44 ± 0.015	0.41 ± 0.015	0.69 ± 0.01	0.61 ± 0.01	0.66 ± 0.015
**24**	0.11 ± 0.001	0.26 ± 0.02	0.15 ± 0.025	0.46 ± 0.01	0.42 ± 0.006	0.47 ± 0.006
**36**	0.0 ± 0.0	0.0 ± 0.0	0.0 ± 0.0	0.16 ± 0.006	0.11 ± 0.01	0.14 ± 0.006
(**II**) ***S. typhimurium***						
**0**	1.0 ± 0.0	1.0 ± 0.0	1.0 ± 0.0	1.0 ± 0.0	1.0 ± 0.0	1.0 ± 0.0
**6**	0.77 ± 0.02	0.79 ± 0.01	0.76 ± 0.01	0.92 ±0.015	0.87 ± 0.02	0.94 ± 0.015
**12**	0.49 ± 0.02	0.47 ± 0.025	0.42 ± 0.01	0.82 ± 0.02	0.56 ± 0.01	0.79 ± 0.01
**24**	0.23 ± 0.025	0.26 ± 0.025	0.15 ± 0.025	0.59 ± 0.01	0.34 ± 0.015	0.57 ± 0.01
**36**	0.0 ± 0.0	0.0 ± 0.0	0.0 ± 0.0	0.38 ± 0.015	0.18 ± 0.015	0.36 ± 0.015

## Data Availability

The data presented in this study are available on request from the corresponding author.
